# Dehydration survival of crop plants and its measurement

**DOI:** 10.1093/jxb/erx445

**Published:** 2018-01-08

**Authors:** Abraham Blum, Roberto Tuberosa

**Affiliations:** 1 Plantstress.com, Tel Aviv, Israel; 2 Department of Agricultural Sciences, University of Bologna, Viale Fanin, Bologna, Italy

**Keywords:** Breeding, desiccation tolerance, drought resistance, drought stress, *Eragrostis tef*, gene expression, method, mortality, phenotyping, water status

## Abstract

Dehydration survival under drought stress is defined in this review as the transition from plant activity into a quiescent state of life preservation, which will be terminated by either recovery or death, depending on the stress regime and the plant’s resilience. Dehydration survival is a popular phenotype by which functional genomics attempts to test gene function in drought resistance and survival. The available reports on phenotyping and genotyping of dehydration survival in genomic studies indicate that the measurement of this trait is often biased to the extent that misguided interpretations are likely to occur. This review briefly discusses the physiological basis of dehydration survival in resurrection plants and crop plants, and concludes that in phenotyping dehydration survival there is a need to distinguish between dehydration avoidance and dehydration tolerance (also termed desiccation tolerance) in affecting survival and recovery. Without this distinction, functional genomics studies of the trait might be biased. Survival due to dehydration avoidance is expressed by the capacity to maintain a relatively high plant water status as the plant is desiccated. Survival due to dehydration tolerance is expressed by delayed mortality (mortality at a relatively low plant water status) as affected by the resilience of plant metabolism. The common test of dehydration survival, using the relative recovery after a given number of stress days, is therefore insufficient because it is mainly driven by dehydration avoidance and so ignores a possible role for dehydration tolerance. Conceivable methods for more accurate phenotyping of the two components of dehydration survival are proposed and discussed.

## Teaser

Dehydration survival depends on dehydration avoidance and/or tolerance. The responsible factor is often not recognized in tests of dehydration survival. Possible correct methods for assessment of dehydration survival are proposed.

## Introduction

Dehydration survival under any stress is defined here as the transition from plant activity into a quiescent state of life preservation, which is terminated by either recovery or mortality depending on the stress regime and the plant’s resilience. Research reports on the breeding or genomics of drought resistance do not always make a clear distinction between drought resistance as a general expression of adaptation to drought stress via signal-dependent responses or constitutive capacity on one hand, and the inactive quiescent state of survival on the other hand.

The terminology that will be used in this review for drought stress and plant adaptation, as discussed by Blum (2016), requires brief clarification. A plant’s response to drought stress depends on the specific strain caused by stress in the plant. For example, stress can cause a dehydration strain, or a hormonal strain or signal, which might be regulated by dehydration (Wilkinson and Davies, 2002; Farrant and Ruelland, 2015). The overall plant response/adaptation to dehydration strain is divided into two major pathways: dehydration avoidance and dehydration tolerance (also termed desiccation tolerance). Here, we will address dehydration survival under dehydration strain, as it can be driven by the plant’s capacity for dehydration avoidance and/or dehydration tolerance and the gene regulation underlying these respective capacities. Survival due to dehydration avoidance is manifested as the capacity to maintain a relatively high plant water status, measured as, for example, relative water content (RWC), as the plant dehydrates. Survival due to dehydration tolerance, in contrast, is expressed by delayed mortality (i.e. mortality at a relatively low plant water status) driven mainly by a more resilient plant metabolism, a feature typical of resurrection plants (discussed below).

The capacity to survive dehydration is an important evolutionary component of plant life in harsh environments. While survival is the most basic feature of resurrection plants, it can sometimes also be an important component of crop adaptation in extreme environments. Plant production, rather than mere survival, is the major consideration in modern economical dryland farming (Passioura, 2006; Blum, 2011), but the dehydration survival of food plants and forage crops can sometime also determine human survival in harsh subsistence farming. Dehydration survival is also ecologically important in forests, natural perennial plants, and pastures. Yet, dehydration survival is not a major trait in modern crop plant breeding, especially as crops enter their reproductive growth stage at a point where it is virtually impossible for them to recover from reproductive failure. Furthermore, even a high capacity for survival under prolonged drought stress is likely to cause a serious delay in the phenology and seasonality of the crop: the return to crop growth upon recovery might place it outside its normal season, leading to possible problems of temperature stress, biotic stress, and/or difficulty with harvesting.

Dehydration survival has become a common phenotypic expression by which genomics research, and sometimes certain breeding programs, test for ‘drought resistance’ in the laboratory. This notwithstanding, plant survival is not necessarily a measure of a crop’s drought resistance in the field (Skirycz *et al.*, 2011), where stress and adaptation interact with growth, plant developmental phenology, and physiology. However, if the purpose of these laboratory tests is just to predict dehydration survival capacity, then such protocols should recognize and properly dissect the real basis of survival and recovery, especially where functional genomics is involved.

This review will propose a logical way to perform the phenotypic dissection of dehydration survival, which should also better associate the survival phenotype in the laboratory or the field with the genes expressed in a laboratory test, particularly when dealing with crop species. Accordingly, this is neither an extensive review of resurrection plants nor a review of novel findings in these species, but rather a summary of what we can learn and possibly utilize from their capacity for dehydration survival, in the hope of designing better and more meaningful phenotyping methods which will eventually facilitate identification of the key genes whose expression is able to increase the dehydration survival capacity of crops.

## The physiological basis of dehydration survival

### Resurrection plants

Survival in states of extreme desiccation (i.e. dehydration) is the foundation of the evolutionary success of resurrection plants. Certain resurrection plants can recover from plant moisture content similar to that of viable seeds. In many cases, research on resurrection plants has been justified as a possible step towards engineering the resurrection trait into dryland-adapted forage and crop plants.

Costa *et al.* (2016) argued that the development of desiccation-tolerant life forms in general, such as seed and pollen, must have been accompanied by the acquisition of dormancy or a dormancy-like state, and that this is also expressed in resurrection plants. The progression into survival dormancy may involve the maintenance of cell homeostasis, formation of intracellular glass, accumulation of protective sugars, late embryogenesis abundant (LEA) proteins, and antioxidants, protection against mechanical stress, maintenance of cell skeleton integrity, and cytochrome P450 family members (Farrant *et al.*, 2012; Giarola *et al.*, 2017). The conservation of the photosynthetic apparatus in dry resurrection plants is a particularly notable and intriguing feature, as these plants recover upon rehydration and return to CO_2_ fixation. When *Craterostigma pumilum* plants are dehydrated, a specific order of metabolic events can be observed in the photosynthetic thylakoid membranes, which helps to prepare the plant for the desiccated state and minimize the production of reactive oxygen species (ROS) (Zia *et al.*, 2016). Transcript profiling has shown that several hundred genes might be differentially expressed in response to dehydration (Rodriguez *et al.*, 2010). Bartels and Salamini (2001) concluded that the basic patterns of changes in gene expression that occur in response to dehydration can be summarized for *Craterostigma plantagineum* as follows: (i) some transcripts accumulate to high levels during dehydration, and disappear early during rehydration; (ii) some transcripts accumulate transiently during the initial dehydration phase; (iii) some transcripts decline during dehydration; and (d) some transcripts remain unchanged in response to dehydration. Studies of tissue-specific expression patterns and subcellular localization have revealed specific cellular distributions of RNAs and proteins that appear to correlate with their predicted functions (Phillips *et al.*, 2002).

While some features of resurrection plants are constitutive, such as their high cellular sugar content, their high dehydration tolerance largely depends on gene expression in response to dehydration strain. It is therefore clear that transcript accumulation and gene expression in resurrection plants are highly responsive to the rate and level of tissue dehydration, similar to what has been shown in crops (Talamè *et al*, 2007; Habash *et al.*, 2014; Zhang *et al.*, 2014). The rate of dehydration is widely recognized as an important factor in dehydration tolerance in the plant kingdom, evidently because adaptation processes take time and might also be a function of the level and timing of dehydration reached. For example, the acquisition of dehydration tolerance in *Sporobolus stapfianus* requires desiccation to at least 60% RWC (Griffiths *et al.*, 2014). The onset of osmotic adjustment in rice (*Oryza sativa*) requires a reduction of RWC to at least 70% (Babu *et al.*, 1999). Work with *Boea hygrometrica* (Mitra *et al.*, 2013) revealed that it adapts well under fast drying only if acclimated under previous drying cycles.

In their study of desiccation tolerance of *Xerophyta viscosa* (Baker), Farrant *et al.* (2015) proposed a model with three stages of plant responses to dehydration: (i) an early response to drying (ERD) in which RWC declines from full turgor to ~55%, during which leaf color changes from green to yellow, indicative of photosynthetic shutdown; (ii) a late response to drying (LRD) occurring between 55 and 10% RWC, during which leaves fold adaxially and exposed surfaces become anthocyanin rich; and (iii) below 10% RWC, when respiration ceases and tissues eventually reach an air-dry state (ADS) of ≤5% RWC. Farrant *et al.* (2015) also detail the different metabolic components that have been shown to be up- or down-regulated according to these different stages of dehydration. Farrant and Moore (2011) presented a useful schematic graphical model of events and adaptive processes that develop in plants, seeds, and resurrection plants along a gradient of RWC or plant water potential. While it can be argued that a refinement of this scale might be necessary for mesophytes, as an educational tool it remains clear that plant physiological, metabolic, and genomic events are linked to the specific rate of plant dehydration as the plant adapts (Talamè *et al.*, 2007) and approaches survival or apoptosis (e.g. Djilianov *et al.*, 2011). This is the benchmark for assessing any plant response to dehydration, where, regretfully, more than a few drought stress studies fail (Blum, 2014).

### Mesophytes as representatives of crop plants

Most research on the dehydration survival of mesophytes has been done in natural vegetation, forest trees, and native range species. Much less data is available for crop plants, reflecting the relatively greater ecological importance of survival in natural native vegetation. However, even mesophytes prepare for survival when severe dehydration proceeds, in tune with their limited capacity to withstand and survive low RWC. The higher capacity for dehydration survival shown by resurrection plants is not possible in crop plants, probably because of the inherent tight trade-off between good growth potential and the constitutive or adaptive capacity for extreme survival under severe dehydration (Claeys and Inzé, 2013). For example, Zhu *et al.* (2009) found that *B. hygrometrica* heat shock factor BhHSF1 expressed in Arabidopsis and tobacco regulated growth retardation, which preceded the accumulation of various proteins linked to dehydration tolerance, while negatively regulating cell-division-related genes. Arrested growth is a genetically controlled precondition for good survival (Claeys and Inzé, 2013).

Arrested growth under progressing stress will lead to a dormant or quiescent state; this is a common condition towards survival as also seen in freezing survival of crop plants, winter survival of deciduous trees, and even summer survival of Mediterranean native grasses (Norton *et al.*, 2016). Depending on the type of stress, this dormancy might be signaled by hydraulic, hormonal, or even pH cues (Schachtman and Goodger, 2008); temperature, photoperiod, and seasonality can also be involved. Abscisic acid (ABA) is the key regulator towards a quiescent state under dehydration strain (e.g. Sreenivasulu *et al.*, 2012).

Taken together, the above clearly shows that duplication of the resurrection phenotype in a crop plant cannot be a practical solution for improving crop drought resistance, although certain very specific components of the resurrection system, such as the LEA proteins, could possibly support crop plant drought resistance in terms of productivity and/or survival (Babu *et al.*, 2004; Hussain *et al.*, 2011; Chen *et al.*, 2015; Gürel *et al.*, 2016).

Mortality due to drought stress in mesophytes is generally caused by two major responses: the direct cellular consequences of dehydration/turgor loss and/or carbon starvation. The former is generally more prevalent in herbaceous annuals and most crop plants, while the latter is more common in trees because of their relatively slow and prolonged dehydration (McDowell, 2011). The isohydric plant phenotype, which is more typical of natural vegetation, tends to close stomata at relatively higher plant water status, which may conserve water but also lead in time to starvation-related mortality. Hydraulic failure due to xylem embolism is a common reason for drought-associated mortality in trees (Barigah *et al*, 2013; Gleason *et al.*, 2014), but sometimes both hydraulics and carbohydrate status interact to regulate survival (Savi *et al.*, 2016).

The work of Farrant *et al.* (2015) indicates that the lowest (critical) plant water status at which mesophytes can survive is a RWC of ~40%. For soybean genotypes grown in soil in the ground, the critical RWC at which plants died ranged between 49 and 41%, but time to survival ranged between 27 and 41 days, respectively, indicating a possible role of dehydration tolerance in extending the time to plant death (James *et al.*, 2008).

As a plant approaches its critical plant water status it enters the phase of programmed cell death, of which senescence might be taken as an early expression (Greenberg, 1996). Here, dehydration tolerance becomes important in delaying mortality via some of the mechanisms that are typical of resurrection plants (e.g. enhanced cell membrane resilience or LEA protein accumulation). It follows that dehydration survival can be driven by either dehydration avoidance or dehydration tolerance, or both.

The difference between the two mechanisms of survival is not always recognized. This is demonstrated by the study of Campo *et al.* (2014) in rice; these authors concluded that ‘Compared with control plants, *OsCPK4* overexpressor plants exhibit stronger water-holding capability and reduced levels of membrane lipid peroxidation and electrolyte leakage under drought or salt stress conditions’ (Campo *et al.*, 2014, p. 688). If the mutant differed in water-holding capability and tests for dehydration tolerance factors such as membrane lipid stability and membrane stability were not normalized for plant water-holding levels, then the assumed tolerance advantages of the mutant could be simply ascribed to a difference in dehydration avoidance and not necessarily to tolerance factors such as membrane stability. The same consideration often applies to studies in which genetically engineered plants are evaluated for their resilience to water stress (e.g. Blum, 2014; see also http://www.plantstress.com/Devil/devils.htm). It is therefore extremely important to resolve the two mechanisms when ascribing a function to a gene expressed under drought stress.

## Phenotyping for dehydration survival

Dehydration avoidance appears to be the common basis for dehydration survival and recovery in crop plants (e.g. Blum *et al.*, 1981; Likoswe and Lawn, 2008; Guan *et al.*, 2010; Blum, 2011; Rosales *et al.*, 2012). In the field, dehydration avoidance capacity can generally be driven by means of effective soil moisture capture, osmotic adjustment, reduced canopy albedo, high cuticular hydraulic resistance, stomatal regulation by ABA, and even small plant size. However, not all of these options are available to plants grown in pots under laboratory conditions, where most survival assays are performed. Under these conditions, the most likely driver of dehydration avoidance is osmotic adjustment (Lilley and Ludlow, 1996; Wright *et al.*, 1997) or ABA-induced stomatal closure (e.g. Wilkinson and Davies, 2002).

The corollary for a realistic phenotyping protocol of dehydration survival and its metabolic basis is sufficient time under dehydration strain, namely, slow drying. This is true for native desiccation-tolerant plants such as bryophytes (Cruz de Carvalho *et al.*, 2017) and crops such as sorghum (Jones and Rawson, 1979). Although there is no set prescription, dehydration rate is related to the plant size, available soil volume, and the soil and atmospheric environment, all of which determine the rate of water loss from plants. A very general rule of thumb is at least about a week to the onset of visible wilting, depending on the species. Pot experiments are especially susceptible to fast drying (Poorter *et al.*, 2012).

It is a common practice in phenotyping studies of plant dehydration survival to grow seedlings and score them for mortality (usually visually) or for recovery upon rehydration after a given number of days since the last irrigation. In gene-expression studies, RNA is sampled on that day, and the assumption is made that all genotypes were therefore subjected to the same level of dehydration strain. This protocol can result in artifacts, since genotypes can differ in their water status on the day of sampling; hence, apparent genetic variation for dehydration tolerance as a survival mechanism might be due not to a specific gene effect but to possible variation in dehydration between genotypes. For example, in rice, Zhou *et al.* (2007) identified 301, 448, and 1020 genes to be induced under drought stress in leaf, panicle, and shoot, respectively. It is reasonable to suspect that the different rice organs varied in terms of water status, which was not measured. Therefore, the reported gene numbers could have been driven by differences in water status among the organs.

In attempting to obtain a similar water status in all tested genotypes under dehydration conditions, Norton *et al.* (2016) suggested assessing survival by scoring plants for mortality after a given number of days counted from the day of full stomatal closure. With this method, stomatal closure was taken as an indication of the same level of initial dehydration in all tested genotypes. This is an important step forward even in view of the known involvement of ABA in stomatal closure, but it is a practical improvement over merely counting the number of days without watering until the point of testing. The proposed method might also help to verify whether the plant species in question is an isohydric or an anisohydric plant (Tardieu and Simonneau, 1998). Isohydric plants are more likely to close their stomata at higher leaf water status due to ABA involvement and thus maintain turgor for a longer time with closed stomata. Anisohydric plants are likely to close their stomata in response to a hydraulic signal, namely at loss of turgor. Thus, the suggestion of Norton *et al.* (2016) is more appropriate for anisohydric plants. Sinclair (2000) concluded from his model of dehydration survival that cuticular transpiration can also determine phenotypic variations in survival due to its effect on dehydration. It follows that an accurate dissection of the genetic basis [e.g. quantitative trait loci (QTL)] of the response of plants to dehydration, and their survival, is possible only when all genotypes are phenotyped at the same low plant water status, an issue that is particularly critical under field conditions, where large differences in water status are common in segregating populations, particularly when segregation of major loci for phenology increases variability of flowering (Tuberosa, 2012).

**Fig. 1.**
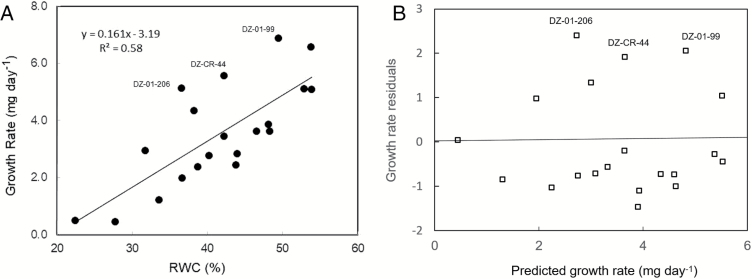
(A) Linear regression of recovery growth (i.e. growth rate after recovery irrigation) on RWC at peak stress (before recovery irrigation) in 20 tef (*Eragrostis tef*) cultivars at the juvenile growth stage. Plants were grown in pots under 18/25 °C night/day temperatures and 480 µmol m^−2^ s^−1^ of photosynthetically active radiation, and irrigated with half-strength Hoagland’s nutrient solution. Irrigation was terminated at 25 days after emergence. Recovery irrigation was applied at 33 days after emergence, when most leaves were wilted or desiccated. Just before recovery irrigation, plants were sampled for their total shoot dry weight and the RWC of the “pseudo-stem”, which consisted of the basal leaf sheaths enfolding the growing meristem. Ten days after recovery irrigation, a second sample of plants was taken for determination of total shoot dry matter for the calculation of daily plant growth rate (Blum 1998, unpublished data). (B) Deviation of tef genotypes from the regression of cultivar recovery growth rate on RWC (shown in A) presented as studentized residuals [the growth rate residuals (outliers of the regression) divided by an estimate of their standard deviation (Pope, 1976)] of actual growth rate compared with the predicted growth rate according to the regression. The three genotypes labeled are those with the most positive deviation.

Fig. 1 illustrates a simple phenotyping method to differentiate between the two possible mechanisms for survival, namely, dehydration avoidance and dehydration tolerance, in different genotypes of tef (*Eragrostis tef*) (Blum 1998, unpublished data). Recovery growth after dehydration was positively and linearly associated across all genotypes with their plant water status at peak stress (R^2^=0.58), that is, their capacity for dehydration avoidance (Fig. 1A). Three of the studied cultivars appeared to be positively deviated from the regression. This could be seen in the studentized deviation of actual recovery growth from the predicted growth (Fig. 1B). This result suggested that these three cultivars might possess recovery capacity based on dehydration tolerance, above and beyond their capacity for maintaining their water status. This method potentially allows discrimination of the existence of the two mechanisms, facilitating their further analysis and genetic dissection based on suitable genotypes with contrasting phenotypes.

It follows that assessment of plant recovery, as is commonly performed after a given number of days of dehydration, may identify survival mainly by way of dehydration avoidance, while the resolution of dehydration tolerance requires normalized RWC at the point of recovery.

The identification of extreme genotypes for tolerance-based dehydration survival allows the development of biparental populations of recombinant inbred lines (RIL) suitable for dissecting the QTLome that governs the water status and growth rate of the plant. Additionally, comparative analysis of the size and direction of the concurrent additive effects of each QTL on multiple traits allows researchers to elaborate hypotheses on the causative relationship between such traits (Lebreton *et al.*, 1995; Tuberosa *et al.*, 2002) and, ultimately, define models that are able to predict crop performance in different environments (Tardieu and Tuberosa, 2010).

Phenotyping tolerance-based dehydration survival requires normalizing for RWC. This entails tracking of RWC in a population of perhaps 200 RILs to be able to test for recovery at standard RWC. Classic tests of RWC by leaf sampling are labor-intensive and will be biased by diurnal fluctuations; hence, alternative surrogate high-throughput methods for phenotyping of RWC should be employed. For example, Woo *et al.* (2008) used remote chlorophyll fluorescence sensing to estimate the dehydration survival of three *Arabidopsis thaliana* accessions. Fluorescence was used to track RWC in the three accessions, and all lost fluorescence at about the same RWC of 20–30%. Other possible methods are thermal imaging and spectral analysis of plants (Fiorani and Schurr, 2013; Araus and Cairns, 2014) with a minimal number of RWC verifications by the classical method. RWC can also be non-destructively estimated and tracked via more sophisticated methods of plant water status tracking in high-throughput systems such as that of Halperin *et al.* (2017).

Once plants are at the standard RWC, for survival testing they can be irrigated and their recovery assessed by means of visual scores and/or estimates of growth. The visual assessment of plant mortality (usually by total leaf desiccation) is subjective and can present problems. For example, all leaves might appear to be dead but meristems can still be alive. Alternative methods for measuring the loss of important life functions can be considered, such as the popular and recommended assessment of cell membrane stability by the electrolyte leakage method. Wang *et al.* (2002) used the ‘critical water status’ when leakage increased sharply to estimate mortality. Plant mortality can also be evaluated by assessing membrane activity (e.g. by the uptake of dyes or the triphenyl tetrazolium chloride test; see below) or, more commonly, through analysis of the metabolome and/or transcriptome profiles (Giarola and Bartels, 2015; Shankar *et al.*, 2016; Giarola *et al.*, 2017) including the expression of apoptosis-related genes (Lam *et al.*, 1999). However, measurement of growth rate upon rehydration (e.g. Fig. 1) is still a popular, simple, and integrative method of assessing plant viability, especially when large plant populations are being phenotyped.

There are two major ways in which plant recovery occurs, which might depend on plant development: (i) when some leaves are still alive, they may recover; or (ii) if all leaves are dead, recovery can proceed from surviving meristems. Ludlow (1980) used leaf water potential at total plant leaf death (visually estimated) as a measure of tolerance. Since osmotic adjustment was a major reason for extended survival in these experiments, Lilley and Ludlow (1996) also used leaf osmotic potential at total leaf death. However, since leaf death is one means by which plants can conserve water in order to extend meristem life (Munné-Bosch and Alegre, 2004), the leaf death score might represent survival via dehydration avoidance and not necessarily tolerance (e.g. Zhou *et al.*, 2009).

If plant dehydration survival via tolerance is to be determined by meristem functionality, the direct measurement of meristem water status remains a challenge. Here, meristems that are axial—such as those allowing recovery by tillers in cereals—may be involved. Work in wheat demonstrated that the capacity of axial meristems to recover by means of tillering diminishes with increasing plant age (Blum *et al.*, 1990). A rough approximation of meristem water status and tolerance in juvenile cereals and grasses (e.g. Volaire and Lelievre, 2001) can be obtained by the measurement of RWC after apparent total leaf mortality; RWC is measured in the basal section of the ‘pseudo-stem’, which consists of the leaf sheaths enfolding the apical meristem. Methods for direct assessment of meristem viability might perhaps involve the application of viability testing methods such as triphenyl tetrazolium chloride staining or other more advanced staining methods.

## Conclusions

Research on dehydration survival has developed to a much greater extent in resurrection plants than in crop plants. The information acquired, and some past research with crops, indicates that dehydration survival can be expressed as dehydration avoidance and/or dehydration (desiccation) tolerance. The current common methods for phenotyping the dehydration survival of different genotypes, such as measuring plant recovery after a given number of days without watering, do not distinguish between the two components that underlie dehydration, because they mainly assess the dehydration avoidance capacity. Therefore, when such tests do not recognize possible dehydration tolerance they are subjected to misinterpretation, especially when gene expression under dehydration stress is concerned. If dehydration tolerance as a driver of dehydration survival must be assessed, then the response of all tested genotypes for dehydration survival must be normalized for plant water status when plant recovery is phenotyped.

By addressing the basics of dehydration survival, this review offers some insights and guidelines for a logical and relevant phenotyping of this important trait in crop plants.
